# Smartwatch-Detected Atrial Fibrillation on the Morning After a Nocturnal Sleep-Disordered Breathing Notification: A Case Report

**DOI:** 10.7759/cureus.109701

**Published:** 2026-05-26

**Authors:** Toru Maruyama, Michinari Hieda, Mitsuhiro Fukata

**Affiliations:** 1 Cardiology, Haradoi Hospital, Fukuoka, JPN; 2 Cardiology and Nephrology, University of Occupational and Environmental Health, Kitakyushu, JPN; 3 Hematology, Oncology and Cardiovascular Medicine, Kyushu University Hospital, Fukuoka, JPN

**Keywords:** arrhythmia management, cardiology devices, digital health, gerontology, smartwatch monitoring

## Abstract

Smartwatches have been gaining popularity, and smartwatch-based patient-activated recordings have been widely used for screening atrial fibrillation (AF) and sleep-disordered breathing. We encountered a 79-year-old man who routinely activated a smartwatch ECG in the morning. He had documented paroxysms of AF while working as a businessman a decade ago. Thereafter, the paroxysms were not documented after he retired from work; therefore, pulsed field ablation was deferred. However, the smartwatch repeatedly detected AF paroxysms the following morning, only after the smartwatch had issued a breathing-disturbance notification during sleep. Smartwatch ECG cannot completely substitute for conventional ECG, and smartwatch-detected breathing disturbance is not equivalent to polysomnography (PSG)-detected obstructive sleep apnea (OSA). However, this case suggests that nocturnal respiratory disturbance may be associated with daily AF paroxysms and that this possibility may be detected using a single smartwatch. Accumulation of similar cases with simultaneous polysomnographic and ECG monitoring may help clarify the mechanistic relationship between nocturnal hypoxemia and AF paroxysms.

## Introduction

Wearable devices, such as smartwatches, are convenient tools that may help users identify health problems cost-effectively. Health problems such as atrial fibrillation (AF) and sleep-disordered breathing are prevalent in middle-aged and older populations worldwide. Furthermore, the usability of commercially available smartwatches has been reported to be acceptable in EDs [1], for remote patient monitoring [2], and among community-dwelling populations [3]. Herein, we report a case of daily AF paroxysms associated with increased nocturnal breathing disturbances, both of which were documented by a single commercially available current smartwatch model. The possible link between AF and nocturnal breathing disorder is highlighted.

## Case presentation

**Figure 1:**
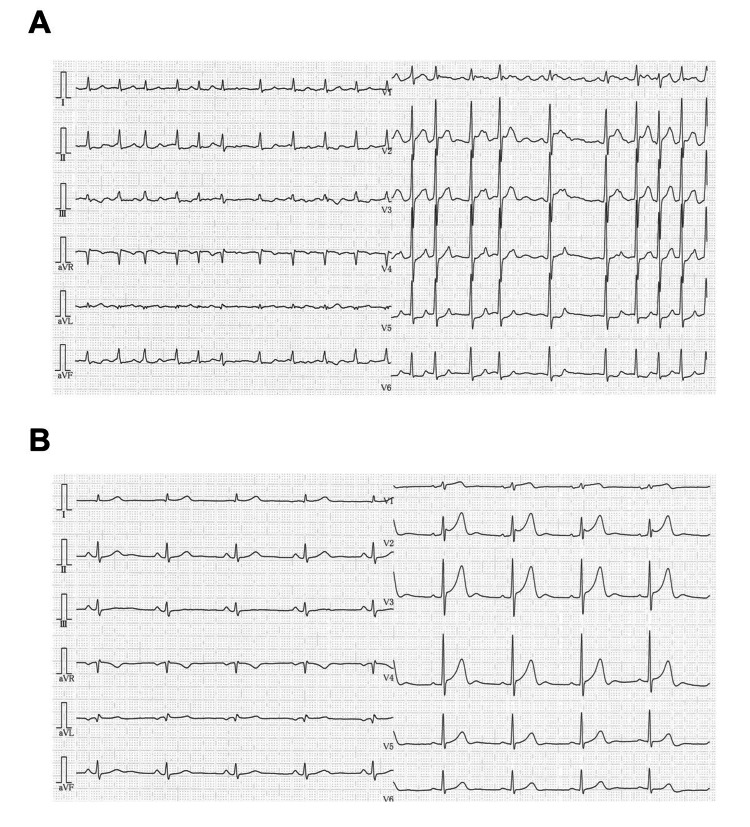
Twelve-lead ECGs recorded a decade ago when the patient complained of palpitations (A) and last year during a routine examination when he was asymptomatic (B). Paroxysmal symptomatic atrial fibrillation (AF) is shown in panel A but not in panel B.

This case report concerns a 79-year-old Japanese man with a history of hypertension, dyslipidemia, type 2 diabetes, and symptomatic paroxysmal AF documented by a 12-lead ECG a decade ago (Figure 1A). He had previously worked as a businessman. Thereafter, he retired from the company and enjoyed his daily life with his wife. His outpatient medications included bisoprolol (5 mg), azilsartan (20 mg), long-acting nifedipine (20 mg), rosuvastatin (5 mg), metformin (750 mg), and a tablet combining empagliflozin (25 mg) and linagliptin (5 mg). Transthoracic echocardiography revealed a left atrial volume index of 28.7 mL/m² (normal range: <34) and a left ventricular ejection fraction (EF) of 55.9% (normal range: >50%), estimated using the modified Simpson method. Blood pressure, glycemic status, and lipid profile were controlled with medication and lifestyle management after retirement. Blood chemistry in the outpatient clinic showed normal hepatorenal function, except for an estimated glomerular filtration rate of 54.0 mL/min/1.73 m² (normal range: >90). Serum lipid levels, reflecting intensive medication, showed a satisfactory profile: total cholesterol of 156 mg/dL (normal range: 142-248), low-density lipoprotein (LDL) cholesterol of 70 mg/dL (normal range: 65-163), and high-density lipoprotein (HDL) cholesterol of 66 mg/dL (normal range: 38-90). The HbA1c (National Glycohemoglobin Standardization Program (NGSP)) level was 6.5% (normal range: 4.9-6.0%), and the NT-proBNP level was 1294 pg/mL (normal range: <55 pg/mL), suggesting heart failure with preserved EF (HFpEF). The combination of bisoprolol, azilsartan, and empagliflozin may have contributed to the biomarker-guided management of HFpEF. AF paroxysms were not documented by ambulatory monitoring or standard ECG (Figure 1B), and we deferred pulsed-field ablation for his AF paroxysms.

**Figure 2:**
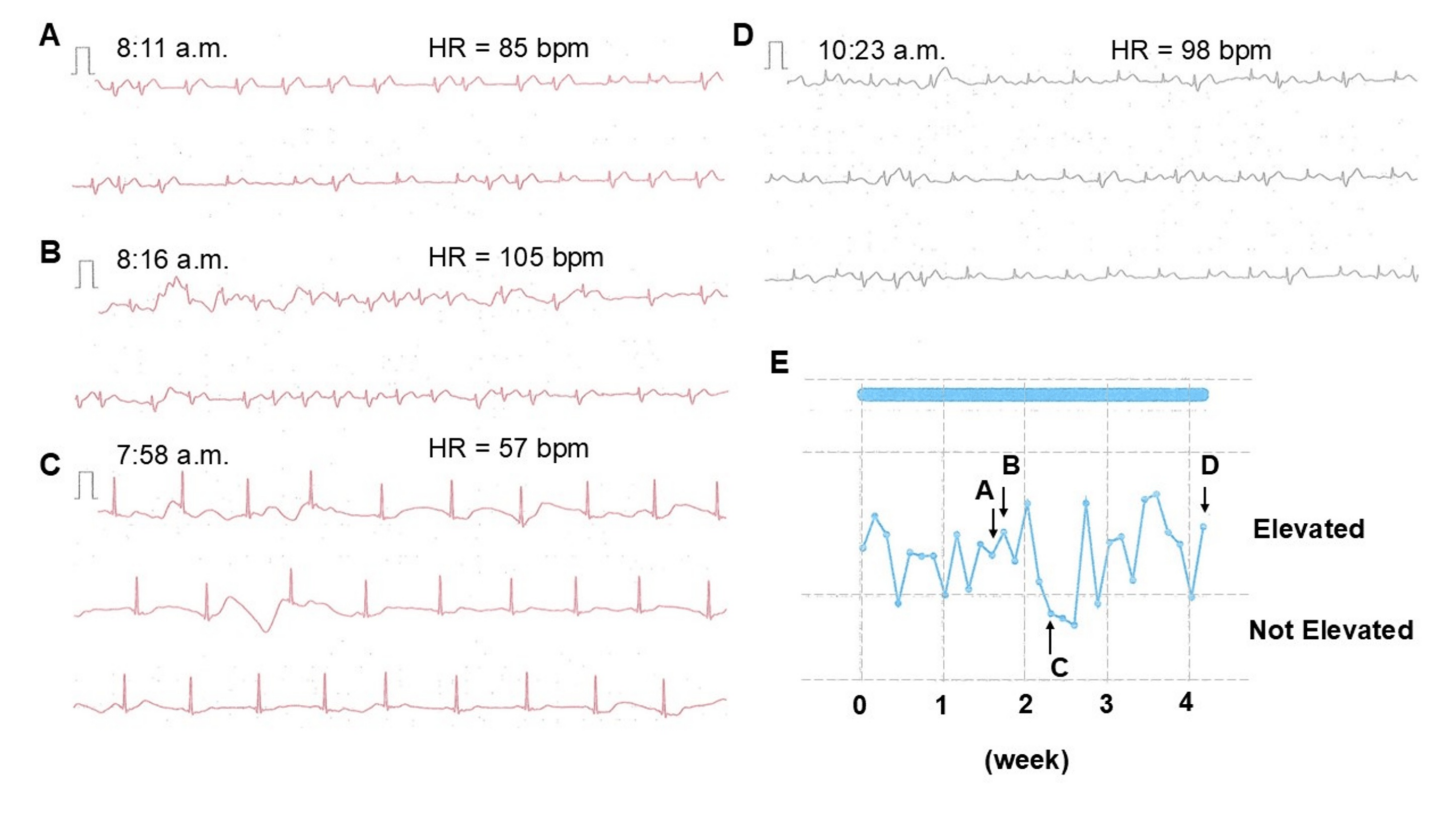
The smartwatch single-lead ECG, activated by the patient and stored on his iPhone each morning, demonstrated AF paroxysms when the “breathing disturbance” level was “elevated” (A, B, and D), but not when it was “not elevated” (C). The weekly trend of “respiratory disturbance” is shown in panel E, and points A, B, C, and D are indicated by vertical arrows. The abscissa represents the weekly time scale, and the ordinate represents breathing status. “Elevated” breathing disturbances may suggest possible moderate to severe sleep apnea; however, confirmation requires polysomnographic monitoring. The Japanese text on the iPhone screen has been translated into English for clarity. AF: Atrial fibrillation; HR: Heart rate.

Several months earlier, he had started daytime recreation for a healthy lifestyle and began using the latest version of the leading smartwatch (Apple Watch Series 11, Apple Inc., Cupertino, CA, USA), paired with an iPhone (iPhone 15 Pro, Apple Inc., Cupertino, CA, USA) via Bluetooth. A few months after starting smartwatch use, he consulted us regarding smartwatch notification alerts related to nocturnal “respiratory disturbance.” The smartwatch data presented on the day of consultation are shown in Figure 2. He activated the smartwatch ECG every morning and evening, irrespective of the nocturnal “respiratory disturbance.” AF paroxysms were detected repeatedly in the morning after notification of nocturnal “elevated” breathing disturbance, but not in the morning after nights when breathing disturbance was “not elevated” (Figure 2), and the morning paroxysms always terminated by evening. He is currently undergoing continuous positive airway pressure (CPAP) therapy, and pulsed-field ablation will be considered depending on the effectiveness of CPAP therapy on the AF paroxysms. A sophisticated multifunctional smartwatch demonstrated promising results in the early detection of recurrent AF paroxysms in a patient with nocturnal breathing disturbance, which was also monitored by the same device.

## Discussion

Recent advances in technology have allowed close monitoring of multiple health data using wrist-worn wearable devices, such as smartwatches. In particular, self-recorded, single-lead ECG is a typical smartwatch-based cardiac health monitoring method that has recently focused on the detection of paroxysmal AF. The diagnostic accuracy of representative smartwatches compared with 12-lead ECG is excellent, with sensitivity and specificity of approximately 95% across multiple systematic reviews and meta-analyses [4-6]. Following the landmark Apple Heart Study [7], subsequent randomized controlled trials have provided evidence that smartwatch-based AF screening can enhance the detection rate of new-onset AF [8], guide anticoagulation [9], and refine the AF diagnostic algorithm [10]. Night-to-night variability in obstructive sleep apnea (OSA) severity underlies the daily variance in AF paroxysms, and this relationship has been confirmed by simultaneous polysomnography (PSG) and ECG monitoring [11]. This case presents data compatible with a close relationship between daily AF paroxysms and nocturnal respiratory disturbance using a single wearable device.

Temporary interruptions of nocturnal breathing cause unintentional restless body motion, and the latest version of the smartwatch tracks these movements using a triaxial accelerometer (X, Y, and Z). Therefore, it can notify users of disturbed breathing patterns during sleep. After continuous nighttime monitoring, the smartwatch evaluates the breathing condition at night as “elevated” or “not elevated” breathing disturbances. “Elevated” breathing disturbances may suggest moderate to severe sleep apnea, which has been validated in studies using simultaneous PSG or other reference sleep testing [12]. Therefore, the U.S. FDA cleared this feature as an over-the-counter device to assess the risk of sleep apnea in 2024 (510(k), Number K240929) [13,14].

Many risk factors for sleep-disordered breathing have been reported, including high BMI, older age, and male sex. This nocturnal breathing disorder is associated with several comorbidities, including hypertension and diabetes. The reported case had diabetes and hypertension, which were well controlled. The main cause of nocturnal respiratory disorder in slender elderly male patients, as in this case (body weight of 65.6 kg, height of 171 cm, and BMI of 22.4 kg/m²), is reduced upper airway muscle tone, leading to upper airway obstruction during sleep. He suspected a breathing disorder at night for the first time after starting smartwatch sleep tracking. OSA, as a representative form of sleep-disordered breathing, is an independent risk factor for AF. The general mechanistic link between AF and sleep-disordered breathing is not fully elucidated, but several pathophysiological mechanisms have been proposed, including autonomic nervous dysregulation, intrathoracic negative pressure, oxidative stress, and inflammatory atrial remodeling leading to fibrosis and arrhythmogenesis [15-17]. Paroxysmal AF was associated with elevated respiratory disturbance tracked by the smartwatch in this case (Figure 2E), implying possible involvement of nocturnal hypoxic stress in the development of AF paroxysms.

As an inherent limitation, smartwatch-detected health disorders are not based on conventional medical diagnostic instruments; that is, OSA should be diagnosed by PSG [18], while the algorithm of the smartwatch used in this case notifies users of signs of restless sleep characterized by tiny body movements due to the interruption of normal respiratory patterns. Smartwatch-detected breathing disturbance is not equivalent to the apnea-hypopnea index on PSG. However, this algorithm was reportedly developed using advanced machine learning applied to an extensive OSA dataset. Popular smartwatches have been validated to detect hypoxemia accurately with concurrent monitoring of pulse oximetry as a reference [19], and one of them has been validated to screen for OSA compared with the gold standard PSG [20].

## Conclusions

Smartwatch technology has enabled the monitoring of a wide variety of health data, contributing to the transformation of digital health. Although many smartwatch-derived findings remain preliminary compared with medically obtained laboratory or diagnostic data, the latest versions of popular smartwatches may demonstrate the feasibility of dual monitoring of heart rhythm and nocturnal respiratory disturbance, which requires prospective validation. Continuous smartwatch-based monitoring of AF episodes may facilitate the assessment of potential associations between AF and lifestyle-related factors, including sleep-disordered breathing, and may support clinical decision-making regarding therapeutic strategies for AF. Furthermore, smartwatch technology may encourage users to consult sleep specialists and cardiologists regarding this specific type of AF.
